# Second-hand smoke in four English prisons: an air quality monitoring study

**DOI:** 10.1186/s12889-016-2757-y

**Published:** 2016-02-04

**Authors:** Leah R. Jayes, Elena Ratschen, Rachael L. Murray, Suzy Dymond-White, John Britton

**Affiliations:** 1 UK Centre for Tobacco and Alcohol Studies and Division of Epidemiology and Public Health, University of Nottingham, Clinical Sciences Building, Nottingham City Hospital, Nottingham, NG5 1PB, UK; 2 National Offender Management Service, Clive house, 70 Petty France, London, SW1H 9EX, UK

**Keywords:** Smoking, Passive smoking, Air pollution, Tobacco, Prison, Smoke-free

## Abstract

**Background:**

To measure levels of indoor pollution in relation to smoking in four English prisons.

**Methods:**

TSI SidePak AM510 Personal Aerosol Monitors were used to measure concentrations of particulate matter less than 2.5 μm in diameter (PM_2.5_) for periods of up to 9 h in selected smoking and non-smoking areas, and personal exposure monitoring of prison staff during a work shift, in four prisons.

**Results:**

PM_2.5_ data were collected for average periods of 6.5 h from 48 locations on 25 wing landings where smoking was permitted in cells, on 5 non-smoking wings, 13 prisoner cells, and personal monitoring of 22 staff members. Arithmetic mean PM_2.5_ concentrations were significantly higher on smoking than non-smoking wing landings (43.9 μg/m^3^ and 5.9 μg/m^3^ respectively, *p* < 0.001) and in smoking than non-smoking cells (226.2 μg/m^3^ and 17.0 μg/m^3^ respectively, *p* < 0.001). Staff members wore monitors for an average of 4.18 h, during which they were exposed to arithmetic mean PM_2.5_ concentration of 23.5 μg/m^3^.

**Conclusions:**

The concentration of PM_2.5_ pollution in smoking areas of prisons are extremely high. Smoking in prisons therefore represents a significant health hazard to prisoners and staff members.

## Background

Second-hand smoke (SHS) causes a range of harmful health effects including lung cancer, lower respiratory tract infections and cardiovascular disease; and exacerbates asthma [1–3]. Awareness of these effects has led governments in the UK and many other countries to introduce smoke-free legislation, and in England, legislation requiring all enclosed work and public places to become smoke-free came into force in July 2007 [4]. The significant reductions in exposure to SHS that this and similar legislation has achieved [5] has resulted in marked reductions in episodes of both cardiovascular and respiratory disease [6–8].

The English legislation did however provide some exemptions, one of which applied to prisons. Prison Service Instruction (PSI) 09/2007 enabled prison Governors in England to make landings and/or wings in prisons smoke-free, but allowed prisoners aged over 18 to smoke in single cells or in cells shared with other smokers [9]. Since around 80 % of the approximately 85,000 prisoners currently detained in England and Wales smoke [10], levels of SHS in some indoor prison areas are likely to be very high, resulting in a significant potential hazard to prisoners, prison staff and visitors.

The concentration of airborne particulate matter <2.5 μm in diameter (PM_2.5_) is a well-established marker of indoor SHS concentrations [11, 12], and previous studies have shown high PM_2.5_ concentrations in environments where smoking has taken place [12, 13]. Although there is no safe level of SHS, standards for indoor air quality produced by the World Health Organisation (WHO) recommend that PM_2.5_ concentrations alone should not exceed 25 μg/m^3^ as a 24 h mean, or 10 μg/m^3^ as an annual mean [14]. Evidence to date on the concentration levels of particulate matter in prisons is limited however [15–17], with little information on ambient concentrations on wing landings or smoking cells, and to our knowledge, no data from prisons in England. This study was therefore carried out to measure PM_2.5_ concentrations, as a proxy measure for second-hand smoke, on prison landings and in smoking and non-smoking cells; and by ambient monitoring as a measure of personal exposure of staff working in these settings.

## Methods

### Prisons

Data were collected from four English Prison Service establishments selected to provide variety in relation to security level, prisoner gender, structural design and size (Table 1). All four prisons had a no-smoking policy for staff members within the prison perimeter, though one had designated areas within the prison grounds for electronic cigarette use by staff members. Prisoners were only allowed to smoke in their prison cell with an exception of one prison which permitted smoking in the exercise yard over lunch periods for those who left the wing all day to work. All had smoke-free wings which included smoke-free cells (Table 1).

**Table 1:** Prison facility characteristics

	Category and function^a^	Structural design	Roll count	Wings	Smoke-free wings	Sampled
HMP 1	Female Closed Local	Built 1960s. Mix of original, T-shaped and quick build wings	262	7	Mother & Baby Unit	July 2014
HMP 2	Male Category C Training	Built 1960s. Mix of triangular, T-shaped and quick build wings	494	8	Care & Separation Unit	August 2014
HMP 3	Male Category B Local	Built 1850s. Victorian radial design	533	7	Healthcare	August 2014
HMP 4	Male Category B Local	Built 1992. Bullingdon design, with additional mix of wings	1215	9	Healthcare & 1 Smoke-Free Spur	October & November 2014

^a^Category B prisons hold prisoners for whom the very highest conditions of security are not necessary but for whom escape must be made very difficult

^a^Category C prisons hold prisoners who cannot be trusted in open conditions but who do not have the resources and will to make a determined escape attempt

^a^Female closed prisons can hold category A, B, C prisoners. Due to the smaller female prisoner population, female establishments are categorised into either ‘closed’ or ‘open’

^a^Local prisons serve the courts and receive remand and post-conviction prisoners prior to their allocation to other establishments

^a^Training prisons hold sentenced prisoners who tend to be employed in a variety of activities such as prison workshops, education and in offending behaviour programmes

### Particulate pollution

PM_2.5_ concentrations were measured using a battery-operated SidePak Personal Aerosol Monitor AM510 (TSI Inc, MN, USA) fitted with a PM_2.5_ impactor and set to a calibration factor of 0.30, as established in the literature to measure tobacco smoke [18, 19]. In accordance with manufacturer’s instructions, SidePak devices were cleaned, the impactor re-greased, zero-calibrated and the flow rate set at 1.7 l/min before each use. PM_2.5_ measurements were logged at one minute intervals, with each one minute data point being an average of 60 s of sample measurements.

### Data collection

Data were collected over three to four consecutive days, typically from a Wednesday or Thursday to Saturday, so that sampling took place in both weekday and weekend regimes, and before and after the ‘canteen’ days when prisoners can purchase tobacco or other personal goods (typically Fridays). A researcher trained in the use of air quality monitoring and surveying, with the help of a prison service headquarters staff member, placed the SidePak monitors in static locations on wing landings and in prisoners’ cells, or attached the monitor to wing-based prison staff to collect personal exposure data during parts of their work shifts.

Fixed locations on wing landings were chosen to cover the range of wing designs and function. Monitors were placed as discreetly as possible to avoid disturbing prisoners’ normal behaviour, though wing officers knew where monitors were placed and for how long. The device was usually placed half way down the wing, above head height and away from open outside doors, windows, or cooking equipment. The monitor keypads were locked during sampling. We collected samples on each day for as long as the researcher was allowed access to the wing, and subject to limitations of battery life and in the case of personal monitoring, staff shift patterns. The gentle buzz emitted from the SidePak monitors could not be heard above the surrounding environmental noise during personal and wing sampling. Data on the layout of the wing, prisoner roll count and lock/unlock times were recorded. Prisoners who inquired were informed that we were measuring air quality.

Wing officers were asked to identify smoking and non-smoking prisoners who were suitable to have a SidePak monitor placed in their cell, and these prisoners were then approached by the researcher who explained the study, answered questions and requested written consent. Given consent, the SidePak monitor was generally placed on a shelf or desk at around waist height in the cell. Data on each cell location, the number of prisoners in the cell, their smoking status and the style of the cell window were recorded. Due to the gentle buzz the SidePak monitor makes whilst sampling it was placed in a cool box surrounded by foam padding. Data were typically collected for a few hours over a morning or afternoon period.

Prison Officers working in the prisons were contacted by email in advance of the study visit, or by word of mouth at the time the monitors were placed on wings or in cells, and invited to volunteer to wear a monitor for personal sampling. All who volunteered were given an explanation of the study and asked to provide written consent. We recruited both current smokers and non-smokers. We measured exhaled carbon monoxide with a Smokerlyzer (Bedfont Scientific Ltd) at the start of our monitoring period, and then attached the SidePak monitor to their belt and used a short length of Tygon tubing to sample air from their breathing zone. A second measurement of exhaled carbon monoxide was taken when sampling finished, when the staff members also returned a timed log of their work locations and activities during the data collection period.

### Data analysis

Since the SidePak monitors were usually turned on and off just before and after being placed in the sampling sites we discarded the first and last five minutes of each data record. Each set of sampling data was downloaded from the monitor using Trackpro 4.6.1 software, and transferred to a Microsoft Excel spreadsheet with the corresponding location, cell and staff member data. We then used STATA 13 to generate descriptive statistics including arithmetic means, 95 % confidence intervals, standard deviations, ranges and times of maximum values, and to estimate the proportion of time in which the PM_2.5_ concentration exceeded World Health Organisation (WHO) 24 h mean PM_2.5_ upper limit of 25 μg/m^3^ [14] for each dataset. Although PM_2.5_ data distributions were skewed, we present arithmetic as well as geometric mean figures since the former are used by the WHO to define upper limits. Log-transformed data were used for all t-test comparisons.

## Results

In total 86 datasets were collected from wing landing, prison cells and personal monitoring. Three datasets were discarded because the monitor had been tampered with, leaving 83 for analysis. Prisoner roll count on the wings sampled varied from four to 180. Details of the number of datasets, and arithmetic and geometric mean, median and range for each type of sample location, including a smoking/non-smoking breakdown, are presented in Table 2.

**Table 2:** Summary of data collected from SidePak monitors located on wing landings, prison cells and whilst attached to staff members

	Sample locations		
PM_2.5_	Wing landings	Prison cells^b^	Attached to staff members^a^
Total Datasets (average duration, hours)	48 (6.5)	13 (4.88)	
Arithmetic Mean (μg/m^3^)	40.08	103.1	
Standard Deviation	57.08	237.47	
Range	0 – 1124	0 – 2684	
Median	30.78	27.52	
Geometric Mean (μg/m^3^)	32.57	59.2	
Interquartile Range	16.40 - 35.85	10.49 – 90.63	
Non-Smoking Locations (average duration, hours)	6 (5.18)	8 (5.12)	
Arithmetic Mean (μg/m^3^)	5.90	16.98	
Standard Deviation	2.90	15.46	
Range	0 – 22	1 – 102	
Median	5.71	13.39	
Geometric Mean (μg/m^3^)	5.58	14.88	
Interquartile Range	5.29 – 7.77	6.9 – 25.82	
Smoking Locations^c^(average duration, hours)	42 (6.66)	5 (4.51)	22 (4.18)
Arithmetic Mean (μg/m^3^)	43.87	226.16	23.51
Standard Deviation	58.95	333.08	34.01
Range	1 – 1124	8- 2684	2 – 608
Median	32.86	162.90	19.04
Geometric Mean (μg/m^3^)	35.57*	122.52*	18.57
Interquartile Range	18.9 – 36.97	81.61 – 163.14	11.37 – 18.59

*Two-sample t-test comparing smoking and non-smoking locations, denotes significance (*p* < 0.001)

^a^All staff members sampled worked on locations where smoking was permitted in cells only

^b^All prison cells sampled were located on wings where smoking was permitted in cells only

^c^Smoking locations were those where smoking was permitted in cells only

### Wing landings

**Fig. 1:**
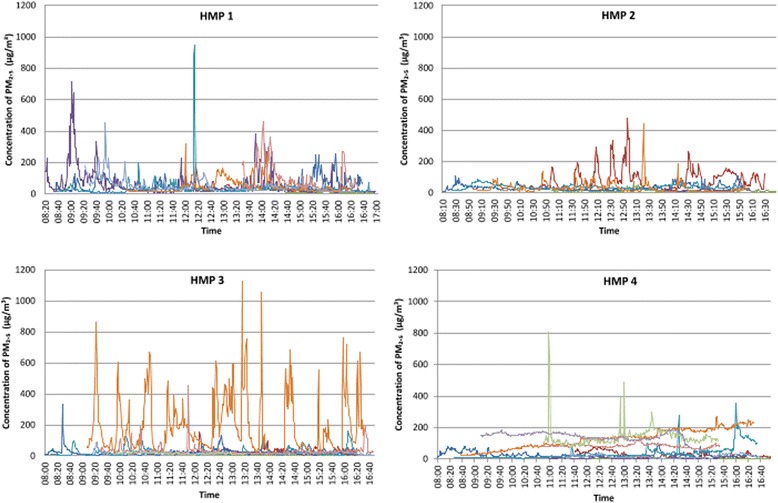
Concentrations of PM_2.5_ recorded on smoking locations in all four prisons sampled over the day time periods

A total of 48 datasets were collected from 30 different smoking and non-smoking landing locations. Thirty-eight locations were sampled exclusively during the daytime period, and ten were sampled into the night time. The average period over which data were collected was 6.5 (Standard Deviation (SD) 2.0) hours. Arithmetic mean PM_2.5_ in the 48 data sets was 40.08 μg/m^3^, and ranged from 0 to 1124 μg/m^3^. Mean PM_2.5_ concentrations were significantly higher on landings where smoking was permitted in cells than non-smoking wing landings (43.87 μg/m^3^ and 5.90 μg/m^3^ respectively, *p* < 0.001). Of the 42 datasets from smoking locations, 18 landings spent over half of the sampling time over the WHO 24 h mean upper guidance limit of 25 μg/m^3^ (14). In the three prisons with a single canteen day (one prison was excluded from the analysis because its canteen delivery spanned two-three days, therefore no pre-canteen data were available), PM_2.5_ concentrations were also higher on smoking locations on the day after the canteen was delivered (20.33 μg/m^3^ before and 27.83 μg/m^3^ after, *p* < 0.001). There was no difference in PM_2.5_ concentrations sampled from wings of different structural design. Continuous data from each smoking site sampled during the daytime are represented graphically in Fig. 1.

**Fig. 2:**
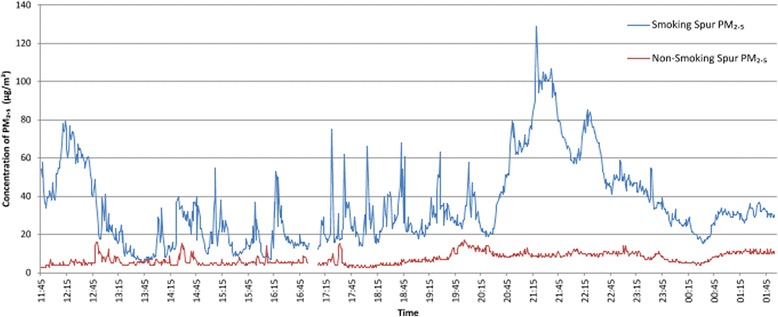
Concentrations of PM_2.5_ recorded on one wing with smoking and voluntary non-smoking spurs

One establishment had a T shaped design wing comprising three identical spurs, one of which was voluntarily non-smoking. The spurs were connected by gated doors which allowed air to flow between them. SidePak monitors were run on the voluntary non-smoking and smoking spur simultaneously throughout the day and then again into the night (Fig. 2).

### Prison cells

**Fig. 3:**
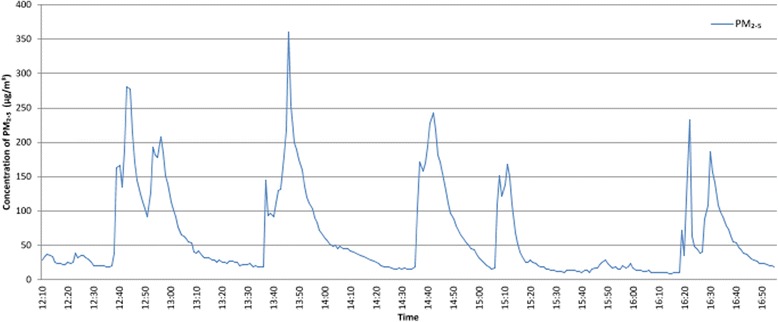
Concentrations of PM_2.5_ recorded in a single smoker cell

All 13 cells sampled were located on wings where smoking was permitted in cells, and five of the cells sampled had occupants who smoked. The average time for which data were collected was 4.88 h (SD 1.76) and the arithmetic mean of the 13 datasets was 103.10 μg/m^3^. High concentrations of PM_2.5_ were recorded in the five smokers’ cells with means ranging from 62.31 to 434.74 μg/m^3^, and in all cases exceeded the WHO limit of 25 μg/m^3^ as a 24 h mean (14) for over 60 % of the sampling time. The arithmetic mean PM_2.5_ concentration in smoking cells (226.16 μg/m^3^) were significantly higher than in non-smoking cells (16.98 μg/m^3^, *p* < 0.001). Figure 3 shows concentrations of PM_2.5_ recorded in a single cell where the occupant smoked. The prisoner reported smoking four hand-rolled cigarettes during the sampling period.

**Fig. 4:**
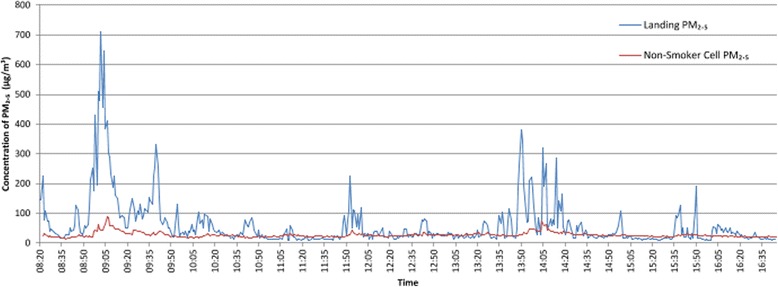
Concentrations of PM_2.5_ sampled simultaneously on a landing and non-smokers cell from the same wing landing

Concentrations of PM_2.5_ in non-smokers cells were relatively low (arithmetic mean 16.98 μg/m^3^), though higher in non-smoking cells on wings with closed narrow corridors than more open designs. Figure 4 shows PM_2.5_ concentrations sampled simultaneously on a wing landing with closed narrow corridors and in a non-smoker’s cell on the same landing. The wing landing had an arithmetic mean PM_2.5_ of 59.78 μg/m^3^ whilst the non-smoking cell located on this landing had a mean of 27.52 μg/m^3^, with concentration levels above the WHO 24 h upper guidance limit almost 50 % of the time.

### Staff members

**Fig. 5:**
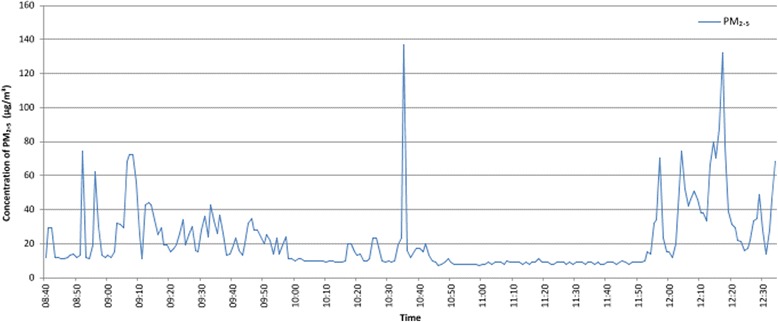
Concentrations of PM_2.5_ sampled during a prison officer’s morning shift. Prison officer self-reported locations and duties during sampling: 08:40–10:00 Wing landing; supervising, dealing with prisoner queries; 10:00–10:10 Wing office; 10:10–11:00 Wing landing; including entering a prisoner cell; 11:00–11:50 Wing office; checking emails and paperwork; 11:50–12:40 Wing landing; supervising lunch time and locking up prisoners

Of the 22 staff members who volunteered for personal monitoring, 21 were prison officers and one a healthcare assistant. All were based on wings where smoking was permitted in cells and had prisoner contact. Twenty-one staff members were monitored during a daytime shift and one on a night shift. The average period of data collection was 4.18 h. The arithmetic mean PM_2.5_ concentration to which participants were exposed was 23.51 μg/m^3^. Figure 5 shows concentrations of PM_2.5_ sampled from a single prison officer during a morning shift alongside their self-reported timed outline of locations and duties during sampling.

The location report for this individual suggested that higher exposure levels tended to occur during periods spent on the wing landings, a finding that was evident in records from all other staff members. Some of the highest concentrations of PM_2.5_ were recorded during duties such as locking or unlocking cells, handing out mail and cell searching. Lower PM_2.5_ concentrations were recorded during periods when staff members were located in the wing office, supervising medication (when the medication hatch was not located on the wing landing) and escorting prisoners off the wing. One prison had 3 staff members exposed to concentration levels above the WHO upper guidance limit of 25 μg/m^3^ [14] for over 80 % of their sampling period.

Carbon Monoxide concentrations in exhaled breath were measured in 21 of the staff members who wore a SidePak monitor. The readings confirmed the smoking status of the staff member participating but did not demonstrate any difference between measures at the start and end of shifts among non-smokers.

## Discussion

This is the first study to measure particulate pollution from SHS in prisons in England. Our findings demonstrate that on wings where smoking was permitted in cells, concentrations of PM_2.5_ sampled on landings and from staff members working on them were high. Although we were for logistical reasons unable to carry out full 24 h monitoring, the concentrations we measured often exceeded the WHO upper guidance limit of 25 μg/m^3^ as a 24 h mean [14], and in some locations did so for the entire period of monitoring. Levels of pollution in cells where smoking was permitted were particularly high. Some of the staff we monitored were exposed above the WHO limit for over 80 % of their working day. Since SHS contains several thousand toxins and many carcinogens [2], the hazards associated with this exposure are likely to be significant. Smoking in prisons is thus a significant potential cause of harm to health in smokers and non-smokers in the prison setting, and including both prisoners and staff.

We used PM_2.5_ concentration as a marker for SHS [11, 12], since direct measurement of tobacco-specific toxins in the atmosphere is expensive and sampling methods would be impractical in prison settings. SHS is not the only source of indoor PM_2.5_, which includes particulate matter released from sources such as open fires, toasters and microwaves. However, where toasters and microwaves were present on the wings, every effort was made to place the SidePak monitors as far away from these as possible. We carried out much of our sampling during the summer months when natural ventilation to the wings and cells through open windows and doors would have been greater than during the winter months, potentially causing our findings to underestimate average pollution levels over the longer term. Safe locations for the SidePak monitors were limited, but we tried to collect data from a broad selection of settings. Since we were obliged to answer questions from staff members and prisoners who enquired about the monitoring, our measurements were not carried out blind. However, whilst it is possible that prisoners or staff changed their behaviour in response to being monitored, we think that is unlikely to have occurred to any appreciable degree over the course of our measurements. Our maximum sampling time was determined by a battery life of around 9 h, though in practice we were also constrained by restrictions on the times that we could leave and collect the monitors. Prison staff who wore monitors were also limited by their shift patterns. For all these reasons our sampling does not provide fully representative 24 h sampling in the prisons; rather it reflects pollution levels at times during the day when prisoners were awake and more likely to be smoking. The proportion of monitoring times spent above WHO guidelines probably therefore overestimates the true 24 h average figures, but the concentration levels observed were at times very high. As a best case scenario, extrapolating the samples from wing locations to cover a 24 h period with an assumption that the times not sampled had a reading of zero, two wings still produced an arithmetic mean above the 25 μg/m^3^ WHO upper guidance limit.

In an evaluation of smoke-free policy within correctional facilities in North Carolina, USA, four facilities with no smoke-free legislation pre-policy recorded an arithmetic mean concentration of PM_2.5_ of 93.11 μg/m^3^ [16]. The arithmetic mean reported for all smoking wing landing datasets in this study is less than half (arithmetic mean 43.87 μg/m^3^) of that reported in North Carolina, even though they report a 65 % prisoner smoking prevalence which, anecdotally, is broadly similar to that in England. Twelve datasets were collected from smoking locations in North Carolina (compared with 42 in this study) and the average time for data collection was 1.28 h (compared to 6.66 h in this study). Another study, conducted in prisons in New Zealand [17] recorded PM_2.5_ concentrations before a smoke-free policy was introduced, and produced a geometric mean before the policy of 6.58 μg/m^3^. Although much lower than the geometric mean recorded across smoking locations in this study (35.57 μg/m^3^) the authors acknowledge that the representativeness of their findings was constrained by their decision, out of fears that the monitors would be tampered with, not to sample air in common areas used by prisoners. Samples were therefore taken only from the ‘staff base’, and did not reflect levels elsewhere in the prison.

Research evidence summarised by the WHO and others suggests that there is no safe level of exposure to SHS [1, 14]. Data collected from staff members gave an insight into locations where exposures to PM_2.5_ were highest, and these included the wing landing, and at the doorway and inside a prisoner’s cell. Taken together, these findings can offer some guidance as to the types of wings or duties where staff members are exposed to the highest levels of SHS and therefore where protection from SHS is particularly needed.

Prisoners in England who want to avoid SHS exposure are entitled to request a non-smoking cell, but our findings suggest that being in a non-smoking cell does not necessarily offer protection against SHS, especially for those on wings with closed narrow corridors. Staff members are also able to opt to work in smoke-free areas of the prison, but such opportunities are relatively rare, resulting in significant exposure for many staff. SHS exposure of pregnant women is also a significant potential hazard [3] for both prisoners and staff members; at the time that this study was carried out, pregnant prisoners were not usually transferred to a smoke-free environment until they have given birth. During data collection at the female closed prison there were 18 pregnant prisoners living on main prison locations, though their smoking status was not known.

Our findings thus provide strong evidence that smoking in prisons in England is a source of high SHS exposure for both staff and prisoners and therefore the current PSI relating to smoking in English and Welsh prisons requires revision. It is likely that our findings are also representative of exposures in similar prison systems in other countries. It is self-evident that this exposure would be reduced by promoting smoking cessation amongst staff and prisoners, increasing the amount of voluntary smoke-free wings and ultimately prevented by making prisons comprehensively smoke-free.

## Conclusions

This is the first study to measure levels of PM_2.5_ as a proxy measure for second-hand smoke in English prisons and demonstrates high levels of smoke pollution in areas of the prisons where people smoke, this therefore represents a significant health hazard to prisoners and staff members. The study provides scientific evidence in support of a national smoke-free prison policy.

### Ethical approval

Ethics approval for the study was provided by the University of Nottingham Medical School Ethics Committee (G06062013 CHS EPH) and the National Offender Management Service National Research Committee (Ref: 2013–202) in July 2014. Permission to enter all four prisons to conduct this study was sought from the Deputy Director of Public Sector Prisons and the Deputy Director of Custody for the South West Area. The four Governors from each establishment also agreed to the research.

### Patient consent and consent to publish

All participants gave informed consent before taking part in the study. This included, consent to publish individual participant datasets.

### Data sharing

Additional data from the study can be obtained on request from the corresponding author.
